# Multi-dimensional-double-spiral (MDDS) inertial microfluidic platform for sperm isolation directly from the raw semen sample

**DOI:** 10.1038/s41598-022-08042-1

**Published:** 2022-03-10

**Authors:** Hyungkook Jeon, Claudia Cremers, Doris Le, Justin Abell, Jongyoon Han

**Affiliations:** 1 Research Laboratory of Electronics, Massachusetts Institute of Technology (MIT), Cambridge, MA 02139 USA; 2 Department of Electrical Engineering and Computer Science, Massachusetts Institute of Technology (MIT), Cambridge, MA 02139 USA; 3 Department of Biological Engineering, Massachusetts Institute of Technology (MIT), Cambridge, MA 02139 USA; 4 Ohana Biosciences, 20 Acorn Park Dr, Cambridge, MA 02140 USA

**Keywords:** Biological techniques, Biotechnology, Medical research, Engineering

## Abstract

Here, we propose a fully-automated platform using a spiral inertial microfluidic device for standardized semen preparation that can process patient-derived semen samples with diverse fluidic conditions without any pre-washing steps. We utilized the multi-dimensional double spiral (MDDS) device to effectively isolate sperm cells from other non-sperm seminal cells (e.g., leukocytes) in the semen sample. The recirculation platform was employed to minimize sample dependency and achieve highly purified and concentrated (up to tenfold) sperm cells in a rapid and fully-automated manner (~ 10 min processing time for 50 mL of diluted semen sample). The clinical (raw) semen samples obtained from healthy donors were directly used without any pre-washing step to evaluate the developed separation platform, which showed excellent performance with ~ 80% of sperm cell recovery, and > 99.95% and > 98% removal of 10-μm beads (a surrogate for leukocytes) from low-viscosity and high-viscosity semen samples, respectively. We expect that the novel platform will be an efficient and automated tool to achieve purified sperm cells directly from raw semen samples for assisted reproductive technologies (ARTs) as an alternative to density centrifugation or swim-up methods, which often suffer from the low recovery of sperm cells and labor-intensive steps.

## Introduction

In 2018, 50–70 million people worldwide were affected by infertility, and about 50% of all infertility cases presented at fertility clinics are caused by male factor infertility and subfertility^1,2^. Of these cases, approximately 60% are due to oligospermia (severe and moderate), a condition characterized by low sperm count^3^. Assisted Reproductive Technologies (ARTs) have been developed to overcome infertility and have enabled the birth of around 3 million children so far^4,5^.

Sperm selection is a central and non-trivial part of ARTs, as the success of the specific ART procedure depends on the quality of sperm used. Therefore, most ART processes (intrauterine insemination (IUI), in vitro fertilization (IVF), and intracytoplasmic sperm injection (ICSI)) require the preparation of the spermatozoa from semen, where motile and morphologically normal spermatozoa are isolated from other cell types (e.g., leukocytes, bacteria, viruses, and dead spermatozoa), debris, and seminal fluid. Especially for IUI, the overall removal of leukocytes and seminal fluid is required, while recovery of large numbers of motile sperm (> 5 × 10^6^) is essential to avoid the decrease in the IUI success rate^6–8^. Although reactive oxygen species (ROS) plays an important role in sperm maturation (e.g., capacitation and acrosome reaction), high concentration of leukocytes in semen samples (> 1 × 10^6^ leukocytes/mL semen) may lead to increased ROS that can damage sperm macromolecules (e.g., DNA, proteins, and lipids) and lead to infertility and affect ART procedure outcomes like IUI^8^.

To prepare sperm cells for ART, cell motility has been almost exclusively used as the primary sperm selection criteria to date, with swim-up (SU) and density-gradient centrifugation (DGC) being the most common preparation methodologies^9,10^. During SU, spermatozoa are self-selected on the basis of their ability to swim against gravity into a fresh culture medium after an initial centrifugation step. DGC can separate sperm cells based on their density and motility. Because motility and morphology affect the actual density of the spermatozoon, motile spermatozoa with normal morphology (> 1.10 g/mL) can be separated from immotile or immature spermatozoa (1.06–1.09 g/mL) as well as leukocytes and cell debris^11^. Therefore, in DGC, motile spermatozoa travel through media with a known density (either one single layer or two layers with defined density, where the top layer has a lower density than the bottom layer) by centrifugation can be recovered at the bottom of the tube^12^. Other used methodologies for cell separation which are rarely used for sperm preparation are fluorescent-activated cell sorting (FACS) and magnetic-activated cell sorting (MACS). Although one can obtain purified motile sperm cells by the current methodologies, they have operational drawbacks requiring laboratory equipment, time- and labor-consuming steps (~ 1 h), as well as well-trained technicians^13–17^. Additionally, there is a critical problem of low overall sperm recovery (e.g., cell loss in the range of 50–70% during DGC^16–18^) and potential cell damage during the separation process^19–22^, which could limit their applicability for ARTs and reduce their success rate, especially in IUI. At least 10 million motile sperm are required to achieve maximum success in IUI^23–25^. Significant loss of sperm in SU and DGC limits the IUI process or causes lower IUI success rates. Furthermore, the motility-dependent separation capability limits the use of the conventional methods to the patients who can only produce immature or low-motility sperm cells^17^.

To overcome the technical limitations of the conventional sperm preparation methods, various microfluidic devices have been introduced^9,13,17,18,21,22,26^. Many of these microfluidic technologies have the drawback of low throughput for sample preparation, allowing just small quantities of sperm to be processed, in the order of a few μL to less than 500 μL, while the number of sperm capable of being processed depends on the dilution condition required for the operation of each microfluidic technology. To overcome this limitation, some inertial spiral microfluidic devices have recently been applied for sperm isolation and successfully demonstrated leukocyte removal with higher throughput and sperm recovery compared to the conventional methodologies^17,18,26,27^. In the inertial microfluidics requiring a high flow rate (order of mL/min) with a finite Reynolds number ($Re = \rho U_{m} D_{H} /\mu$, where $\rho$, $\mu$, and $U_{m}$ represent the density, dynamic viscosity, and maximum velocity of the fluid, respectively, and $D_{H}$ represents the hydraulic diameter of the channel)^28–30^, particle behavior in the microchannel is dominantly affected by inertial lift forces. When the microchannel is not straight but curved (like the spiral device), a recirculating flow called Dean flow arises in the cross-section of the microchannel due to a mismatch of velocity between the fluid in the center and near-wall regions^29^. As a result, in the spiral inertial microfluidic device, lateral particle motion is determined by the balance between inertial lift and Dean drag forces, which enables particle manipulation depending on the particle size. For example, Son et al*.* developed a spiral inertial microfluidic device for sperm preparation, and the spiral device showed 83% of sperm recovery but just removed ~ 90% of leukocyte at a total flow rate of 0.52 mL/min (accumulated from sample and sheath fluid) using washed and diluted seminal cells^17^. Jafek et al*.* introduced another spiral device that showed ~ 90% of sperm recovery with 82% of leukocyte removal at 1.6 mL/min of an input flow rate using washed semen samples. Yet, the drawback of this device is that sperm cells are just loosely focused, not allowing for much sample concentration or reduction of the initial volume^18^. Vasilescu et al*.* introduced a 3D printed inertial microfluidic device and successfully demonstrated the separation of sperm cells from blood cells, epithelial cells, and leukemic cancer cells with high sperm recovery (> 96%)^27^. Although the recent works successfully showed that the inertial microfluidic devices can handle a large volume of a seminal cell sample with relatively high sperm recovery, the separation efficiency is still insufficient and should be improved to increase output quality. More importantly, the effect of the diversity in fluidic conditions of semen (e.g., semen viscosity) on device performance has not been fully appreciated, which is a significant challenge in clinical settings. Seminal cells encompass just a small fraction of semen (~ 5% of volume in semen comes from the epididymis containing the sperm cells)^31^; therefore, it is important to consider other seminal parameters. Washed seminal cells or washed semen samples have been used to determine device performance in all previous works involving inertial microfluidics^17,18,26,27^, which is the main drawback to limit the technology’s clinical impact because the pre-washing step requires manual and time-consuming centrifugation. A useful system for clinical applications should demonstrate high performance regardless of the unique properties of a given (semen) sample without any additional washing steps.

Here, we propose a semen sample preparation platform based on a multi-dimensional double spiral (MDDS) device and a check-valve-based recirculation method^32^. Benefitting from the initial focusing effect in the MDDS device, sperm cells can be effectively isolated from leukocytes with a high separation efficiency and recovery rates (> 90% of sperm cell recovery, > 98% removal of 10-μm beads representing leukocyte, in a single run, using washed semen samples). The separated sperm cells are also further purified and concentrated (up to tenfold compared with the input sperm concentration) by the recirculation platform in a rapid and fully-automated manner (~ 10 min for 50 mL of diluted semen sample). More importantly, directly using clinical (raw) semen samples obtained from healthy donors without any pre-washing step, we demonstrated that the recirculation process offers reliable semen preparation regardless of sample-dependent fluidic properties, i.e., for both for low- and high-viscosity semen samples (~ 80% of sperm cell recovery, and > 99.95% and > 98% removal of 10-μm beads representing leukocytes from the low-viscosity and high-viscosity semen samples, respectively). Considering its separation efficiency and automatability, we expect that the developed platform could be readily deployed in a clinical setting, providing a standardized semen preparation solution not only for ART, such as IUI, but also for other sperm-based analyses and diagnostics.

## Materials and methods

### Fabrication of the semen preparation platform

The MDDS device was fabricated by standard soft-lithographic techniques, as previously reported^32^. A 3D CAD software (SolidWorks) was used to design an aluminum mold with specific channel dimensions, and the mold was fabricated by a micro-milling company (Whits Technologies, Singapore) for standard poly(dimethylsiloxane) (PDMS) device casting. The MDDS device is composed of two sequentially-connected spiral channels with different dimensions; the first spiral channel has a rectangular cross-section of 800-μm width and 60-μm height, and the second spiral channel was designed having larger dimensions and a trapezoidal cross-section with the dimensions of 800-μm width and 80- and 120-μm heights for the inner-wall side and the outer-wall side, respectively. Both spiral channels have three loops, but the trapezoidal cross-section was adapted only for the second spiral channel to increase separation efficiency^33,34^. After making holes in the PDMS replica from the mold for fluidic access by disposable biopsy punches (Integra Miltex), it was irreversibly bonded to a glass substrate by plasma bonding method (Femto Science, Korea). Based on the previous work^32^, the 3D-printed connectors were fabricated by a 3D printer (Form 2, formlabs, USA) using a resin (RS-F2-GPCL-04, formlabs, USA); the channel configuration of the 3D-printed connectors was modified to change the recirculated reservoir from the inner wall outlet to the outer wall outlet. Check valves (80183 and 80184, QOSINA, USA) were used to regulate the flow direction in the recirculation operation; we used the check-valves for single use to prevent potential cross-contamination caused by an internal membrane inside the check-valves. Furthermore, to switch to a single-use disposable medical device, the PDMS MDDS device can be translated to its plastic equivalent that is mass-producible^30^.

### Sample collection and pre-treatment

This study was conducted with ethics approval from the WCG Institutional Review Board (IRB; Study # 1279246). Informed consent was obtained from all subjects, and all methods were carried out following the relevant guidelines and regulations of the IRB. Donors enrolled in the study were between 18 and 45 years old. They were screened for sexually transmitted diseases (STDs), and all were negative. No further information about the semen donors was provided.

Semen samples were obtained by masturbation after 2–5 days of abstinence; the initial semen sample had a volume ranging from 150 µL to 5 mL and a sperm count ranging in 25–150 × 10^6^ cells/mL. Before preparation, samples were liquified for at least 30 min at room temperature (RT). For the initial device characterization, we used washed seminal cells, which were extracted by centrifugation (500 × *g*, 7 min, at RT) of diluted semen 1:5 in modified human tubule fluid (mHTF, Irvine Scientific) with 5-mg/mL bovine serum albumin (BSA, Sigma). Pelleted cells were washed one time in mHTF with 5-mg/mL BSA. In the case that cells needed visualization during the microfluidic operations, cells were transferred into PBS and frozen 15 min at − 80 °C, and then after thawing the cells, they were diluted in PBS containing 1-μg/mL 4′-6-diamidino-2-phenylindole (DAPI, ThermoFisher). Cells can be morphologically unchanged and stained with DAPI more uniformly from the process.

For the experiments of processing clinical (raw) semen samples, semen samples were prepared just by diluting semen in a medium without any pre-washing step. 1 mL of liquified semen was diluted and thoroughly mixed in 5 mL of mHTF with 5-mg/mL BSA, followed by the second dilution step to a final volume of 50 mL also by using mHTF with 5-mg/mL BSA. The two-step dilution process is just for effective dilution and can be simplified by a single-step dilution process.

For DGC preparation of sperm, semen samples were layered on top of a 2-layer IsoLate gradient (IsoLate, Irvine Scientific) and centrifuged for 20 min at 300 × g at RT as per manufacturers specifications. Before further analysis, pelleted sperm were recovered and washed twice in mHTF with 5-mg/mL BSA.

Cryopreserved Human peripheral blood mononuclear cells (PBMCs, ZenBio) were thawed and washed 2 × in PBS before labeling with Cell Tracker (CT) Red (ThermoFisher) as of manufacturer's recommendation. After staining, PBMCs were washed once in PBS and then resuspended in 1-mL mHTF with 5-mg/mL BSA. The PBMC sample was combined with a washed semen sample where sperm cells were stained with DAPI for visualization through the staining process mentioned above. For proper device operation, the mixture was diluted approximately 50-fold in mHTF with 5-mg/mL BSA. Staining the cells enables visualization of the cell trajectory by a fluorescence microscope during device operation and flow cytometric analysis of samples before and after device operation.

### Device operation and sample analysis

The flow rate was regulated by a syringe pump (PHD ULTRA™, Harvard Apparatus, USA and Fusion 200, Chemyx, USA), and trajectories of the fluorescent particles and stained sperm cells were observed by an inverted fluorescence microscope (IX51, Olympus Inc., USA and Axio Observer 7, Zeiss, USA) with a CCD camera (Sensicam QE, PCO, Germany, and ORCA-Flash4.0 V3, Hamamatsu, Japan, respectively). Fluorescent particles in the diameter range of 2–10 μm were used for device characterization; 2.07 μm, 3.87 μm, 4.88 μm, and 7.32 μm (FSFR005, FSEG006, FCFR008, and FSFR007, respectively, Bangs Labs, USA), 3.1 μm, 4.5 μm, and 6.0 μm (19,393–5, 18,430, and 18,141–2, respectively, Polysciences, Inc., USA), and 10.0 μm (F8834, Invitrogen™, USA).

Sample analysis was carried out by flow cytometry (CytoFlex XL, Beckman Coulter). For this, retrieved samples before and after microfluidic processing or DGC were diluted 1:4–1:25 in PBS or mHTF, 5-mg/mL BSA. If cells were initially unstained in the microfluidic device, samples were stained with DAPI (1 μg/mL) and Nuclear-ID red DNA stain (Enzo, live-cell stain, 1:2000). Event analysis was conducted using a flow cytometer (CytoFlex XL, Beckman Coulter).

Sperm motility was assessed using computer-aided sperm analysis (CASA) on a Hamilton Thorne IVOS II using microchambers with 20 μm height (Leja slides 20uM, IMV Technologies). 3 μL of diluted semen (input) and output samples were loaded into the slide. After 1-min rest at 37 °C, sperm motility and composition were analyzed.

### Experimental design

First, to investigate the behavior of human sperm cells in the MDDS device, we used dead, washed human sperm cells stained with DAPI for their observation by a fluorescence microscope. In these initial tests, CT-labeled PBMCs were used as a marker of leukocytes. Various fluorescent beads in the diameter range of 2–10 μm were used to compare their trajectories with the sperm and PBMCs. Movements of cells and beads were observed by a fluorescence microscope under various flow rate conditions, and their recoveries from the outlets of the device were analyzed by flow cytometry. The microscopic observation and quantitative analysis allowed us to determine the behavior of sperm cells in the device, providing identification of the optimal flow rate condition for their isolation from leukocytes. After the initial tests and analysis, (raw) semen samples (not washed seminal cells or washed sperm samples) with various viscosities were tested to demonstrate the applicability of the developed platform in real situations. Here, based on the initial cell and bead trajectory results, fluorescent beads having specific diameters were used to mimic leukocytes^32,35^ and observe the movement of the sperm cells indirectly instead of staining them. Recoveries of sperm cells and beads from outlets were analyzed by flow cytometry, while CASA was used to analyze sperm motility.

## Results

### Isolation of sperm cells from leukocytes by the MDDS device

**Figure 1:**
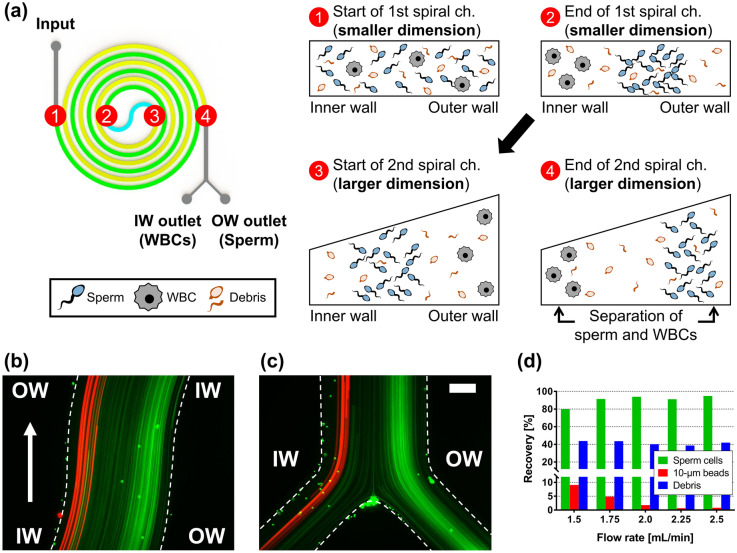
Overview of the semen preparation process by the MDDS device. (**a**) Channel configuration (green: the first spiral channel with smaller dimension, yellow: the second spiral channel with larger trapezoidal dimension) and schematic diagram of the operation process; the first spiral channel has a rectangular cross-section with 800 μm in width and 60 μm in height, and the second spiral channel was designed to have trapezoidal cross-section for the effective particle separation with 800 μm in width and 80 and 120 μm in height for the inner wall side and the outer wall side, respectively. Trajectories of DAPI-stained sperm cells (represented in green color for better recognition) and 10-μm red-fluorescence beads (mimicking leukocytes, represented in red color) at (**b**) the S-shaped transition region and (**c**) the bifurcation region of the MDDS device under the optimal flow rate condition, 2.0 mL/min (scale bar: 200 μm). (**d**) Recoveries of sperm cells, 10-μm beads, and debris from the outer wall outlet depending on various input flow rate conditions. IW, inner wall; OW, outer wall; WBC, white blood cell or leukocyte.

We utilized the MDDS device^32^ to isolate sperm cells from leukocytes, and Fig. 1a shows the channel configuration of the MDDS device and a schematic diagram of the sperm cell isolation process in the MDDS device. The MDDS device is a new type of spiral microfluidic device that our group recently developed^32^, which is composed of two sequentially connected spiral channels having different dimensions. The first spiral channel was designed to have a relatively smaller channel dimension than the second spiral channel to generate a stronger inertial lift force to focus all the input particles into the inner wall side of the channel more effectively (sample focusing); despite the smaller channel dimension, the lift force could not be strong enough for the particles smaller than a specific size, resulting in no focusing or their focusing in the middle of the channel instead of the inner wall side. In the second spiral channel, due to the increased channel dimension, the magnitude of the lift force applied to the particles decreases, and particles move to their equilibrium locations determined by the balance between inertial lift and Dean drag forces, resulting in their separation by size (sample separation). Additionally, to achieve higher separation resolution, the second spiral channel was designed to have a trapezoidal cross-section where a stronger Dean vortex is generated at the outer half of the channel, which leads to more effective expulsion of small particles toward the outer wall without affecting the focusing position of large particles near to the inner wall, resulting in a greater difference between their equilibrium positions^33,34^.

To isolate the sperm cell from leukocytes (or white blood cells) and seminal fluids, we utilized the same MDDS device previously used for leukocyte separation from erythrocytes in human peripheral blood, the details of which can be found elsewhere^32^. The human sperm cell is composed of an ellipsoid head (~ 5 µm in length, ~ 3 µm in width) and a long flexible tail (30–49 µm in length), and in some previous works^26,36,37^, the human sperm cell was assumed to behave as a rotating sphere, with an effective diameter of ~ 5 μm. To investigate the effective size of the human sperm cell and its behavior in the MDDS device, we performed experiments with various fluorescent beads in the diameter range of 2–10 μm as well as sperm and PBMCs and monitored their trajectories at various flow rates in the device, which has the same channel dimensions and configuration with the original device but has 5 outlets for more detailed output analysis (Supplementary Fig. S1a). From these results, we found that the sperm actually behave like spheres in the diameter range of 3–6 μm in the outlet bifurcation region of the MDDS device, and their best focusing and separation from PBMCs can be achieved at a flow rate of 2 mL/min in the MDDS device. Also, behaviors of PBMCs and 10-μm beads at the end of the second channel in the MDDS device were comparable, although leukocyte size significantly varies depending on the subtype (Supplementary Fig. S1b,c)^26,38^. For further experiments, 10-μm beads were used to mimic leukocytes (the leukocyte concentration in normal semen is < 1%, so the addition of beads helps visualize the separation effect). To verify our observation of bead and cell behavior, we mixed sperm cells and 10-µm beads, as shown in Fig. 1b,c the trajectories of sperm cells (green) and 10-μm beads (red) show that both sperm cells and 10-μm beads formed their own focused bands in the different locations of the first channel. 10-μm beads were tightly focused into the inner wall side while sperm cells were less tightly focused, forming a band in the middle of the channel closer to the outer wall. Sperm cells achieved a lower focusing behavior as expected in the first spiral channel, but we found that the initial focusing in the first spiral channel greatly reduced the particle dispersion and helped the 10-μm beads and sperm cells move to their own equilibrium positions with tightly focused bands in the second spiral channel, with sperm focusing on the outer wall side and 10-μm beads focusing at the inner wall side. This resulted in increased separation efficiency compared with the conventional single spiral channel device (Fig. 1a–c vs. Supplementary Fig. S2a,b). As shown in Supplementary Fig. S3, we can obtain highly purified sperm cells by removing 10-μm beads representing leukocytes from the outer wall outlet of the MDDS device. Figure 1d shows the recoveries of sperm cells, 10-μm beads, and debris from the outer wall outlet under various input flow rate conditions. With a flow rate of 2.0 mL/min, sperm cell recovery was > 90%, while the removal rate of 10-μm beads was > 98%. In the case of other debris, which is defined as a separate population having a lower size/density compared to the sperm cells in flow cytometry analysis (Supplementary Fig. S4), the resulting lift force in the spiral channel is not strong enough to focus them to a certain equilibrium position within a focused band, leading to a more even distribution in each outlet. Despite the relatively lower focusing behavior, the flow cytometry analysis of Fig. 1d shows that debris recovery from the outer wall outlet is significantly lower (~ 40% recovery) than the inner wall outlet, which represents that the device also works for debris removal although it is not good enough.

### Sample-dependent performance variation of the MDDS device

**Figure 2:**
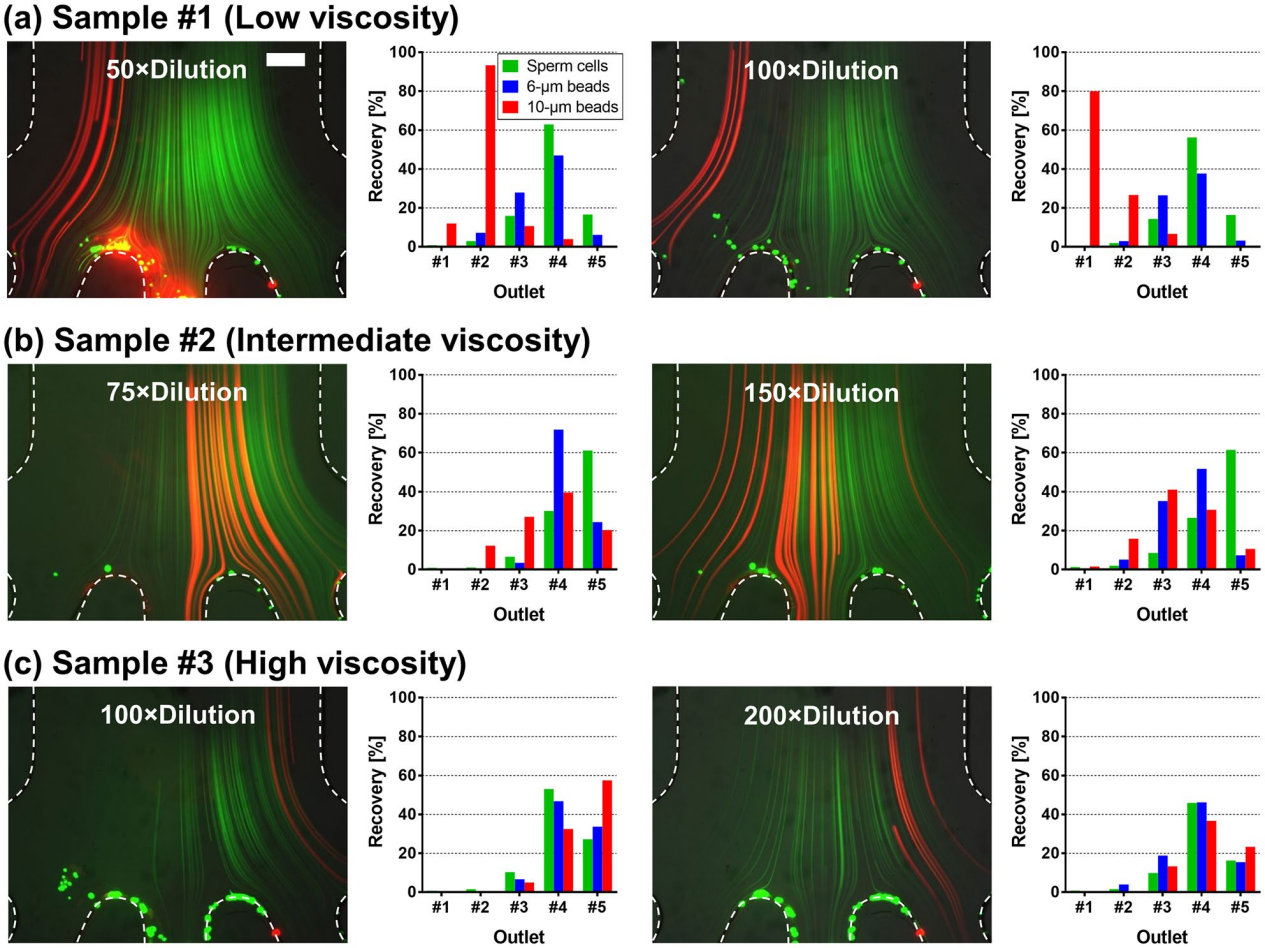
Sample-dependent device performance. (**a**–**c**) Trajectories of 6-μm (green) and 10-μm (red) beads spiked in semen samples having different viscosity conditions (low-, intermediate-, and high-viscosity condition, respectively) with the low and high dilution conditions under the optimal flow rate condition, 2.0 mL/min (scale bar: 200 μm), and graphs showing corresponding recoveries of sperm cells and 6- and 10-μm beads from each outlet; 10-μm beads were used to mimic leukocytes, and 6-μm beads were used to indirectly observe the movement of the sperm cells without staining the sperm cells themselves; viscosity of semen was determined based on WHO guidelines by the thread length when dripping the semen sample (≤ 2 cm for low viscosity, 2–4 cm for intermediate viscosity, and > 4 cm for high viscosity)^41^; mHTF containing 5-mg/mL BSA was used as a buffer solution for sample dilution.

Seminal fluid is composed of secretions from male reproductive organs, including the testes, seminal vesicles, prostate, and bulbourethral gland. Therefore, semen’s fluidic properties (e.g., viscosity) are significantly influenced by individual differences, temporary health conditions, and even ingested foods and water intake^39^. To demonstrate how the change of seminal fluidic properties affects the separation performance of the MDDS device, we observed focusing of the sperm cells and beads from clinical (raw) semen samples (not washed seminal cells or washed semen samples) with various viscosity and dilution conditions in the MDDS device. The device used here has the same channel dimensions and configuration as the original device but has 5 outlets for more detailed output analysis (Fig. 2a–c, Supplementary Fig. S1a). Based on the results that the sperm have similar trajectories with spheres in the diameter range of 3–6 μm, we spiked 6-μm beads into the diluted semen sample to indirectly observe the movement of the sperm cells instead of staining the sperm cells, which could affect cell viability and morphology^37,40^. 10-μm beads were added to mimic leukocytes^32,35^, and modified human tubal fluid (mHTF) containing 5-mg/mL BSA was used as a buffer solution for sample dilution. The semen viscosity was classified based on World Health Organization (WHO) guidelines by its thread length (≤ 2 cm for low viscosity, 2–4 cm for intermediate viscosity, and > 4 cm for high viscosity)^41^. Semen hyperviscosity is not a rare event, an estimated 12–29% occurrence has been reported in men^42^. Therefore, high-viscosity semen should be addressable by a device to be relevant for ART. As shown in the trajectories and the recovery graphs of Fig. 2a–c, sperm cells, and 6-μm beads had similar trajectories regardless of the viscosity and dilution conditions, where the sperm cells were focused to the right-half side in the second spiral channel of the MDDS device so that the majority of them were collected from Outlet #4 and #5. On the other hand, the donor-specific viscosity condition affected the focal positions of 10-μm beads in the MDDS device. As shown in Fig. 2a–c, the trajectories of 10-μm beads moved from the inner wall side to the outer wall side as the semen viscosity increased. Many factors can cause increased semen viscosity, but the exact changes in composition leading to high semen viscosity have not been explicitly identified, and the viscosity may not be homogenous throughout the sample, potentially leading to the formation of an irregular gel-like matrix. However, based on the observation of the microbead behavior, we can assume that the components leading to increased viscosity of the seminal fluids were focused into the inner wall side, preventing the 10-μm beads from achieving their expected equilibrium positions and forcing them towards the outer wall side. Viscosity effects can be reduced by increasing semen dilution mitigating the influence on inertial focusing of particles in the MDDS device. As shown in Fig. 2a–c, better inertial focusing of 10-μm beads was achieved in the higher sample dilution condition (right figures) compared to the lower dilution condition (left figures), although the dilution conditions we tested are still not sufficient to bring back the original trajectory of 10-μm beads and remove them from sperm cells in the samples with an intermediate viscosity and a high viscosity.

### Sample-independent semen preparation by a recirculation platform

Although the viscosity effects can be mitigated by increasing semen dilution, the higher dilution of samples has the drawback of a low-abundant sperm cell output which is not acceptable for ARTs. To avoid this drawback, we recirculated the diluted semen sample to the MDDS device, removing the viscosity-inducing components by recirculation of the outer side sample. Previously, our group developed a recirculation platform based on a check-valve system, where a unidirectional flow is achieved by an internal membrane^32^. A cell solution output from the MDDS separation can be extracted back into the input syringe by the withdrawal motion of a syringe pump and processed again through the same MDDS device repeatedly. This mechanism allows the preparation of samples in large volumes, e.g., 50 mL, to be processed and reduced to a highly purified and concentrated output of 1–2 mL through a series of recirculation. In addition to further purification and concentration, we can minimize sample dependency of separation performance by the recirculation process. As discussed in the previous section, 10-μm beads (representing leukocytes) in the high-viscosity sample were not properly removed from sperm cells during the first circulation process, which might be caused by focusing of the highly viscous, gel-like portions of the semen to the inner wall side. In the recirculation process, a cell solution output from the outer wall outlet is recirculated to the MDDS device, while the output from the inner wall outlet is collected in a waste reservoir. Therefore, from the recirculation process, we can achieve gradual disruption and removal of any seminal fluid material accumulating on the inner wall side so that the viscosity effect can be mitigated, and adequate inertial focusing and removal of leukocytes can be achieved eventually in the remaining cycles.

**Figure 3:**
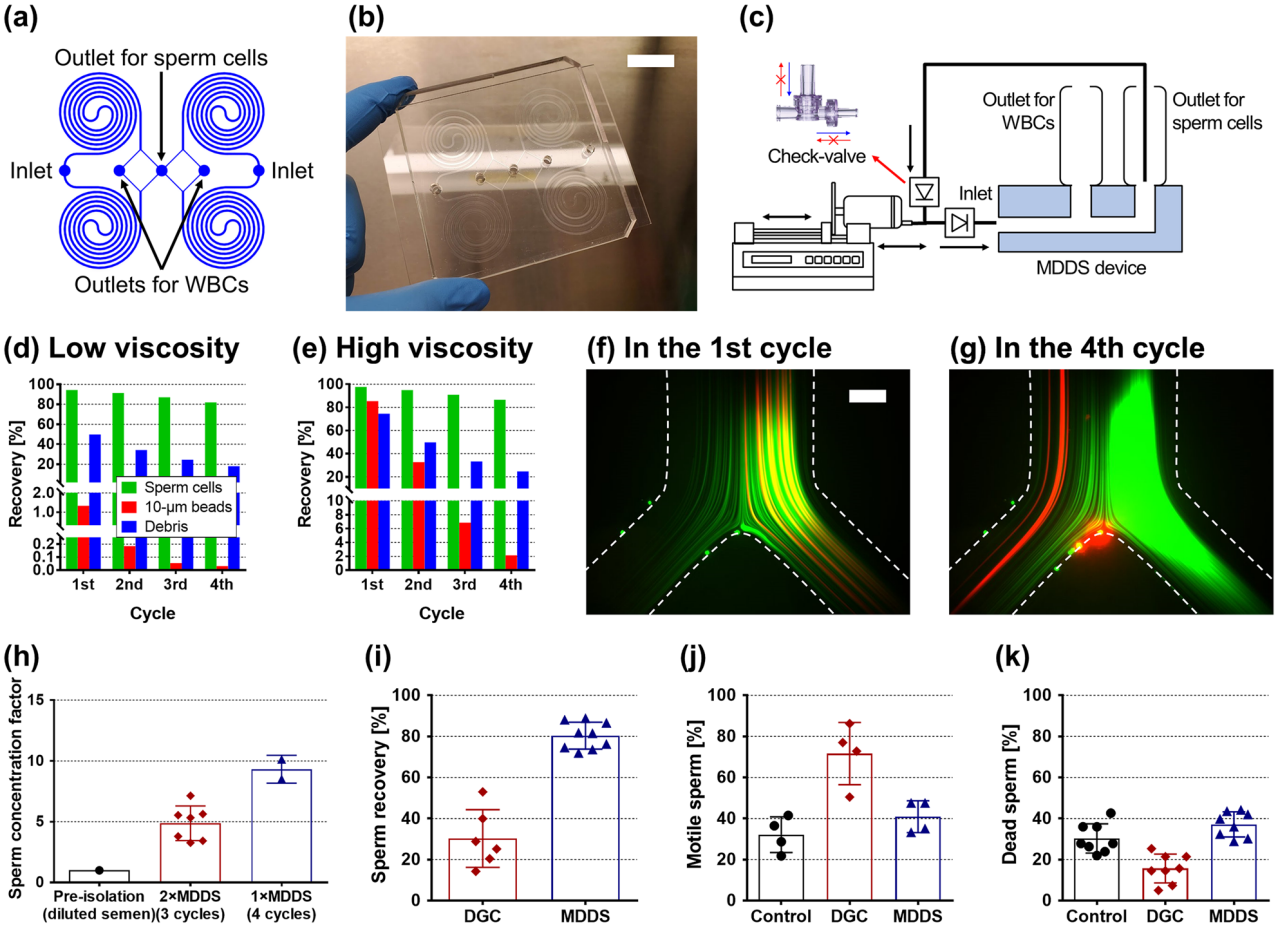
Sample-independent semen preparation process by the recirculation platform. (**a**) Channel configuration and (**b**) a photo of the quad-version MDDS device (scale bar: 2 cm). (**c**) A schematic diagram of the check-valve-based recirculation platform. Recoveries of sperm cells, 10-μm beads, and debris from the outer wall outlet with the 4 cycles of recirculation for the (**d**) low- and (**e**) high-viscosity semen samples under the optimal flow rate condition, 2.0 × 4 = 8.0 mL/min. Trajectories of 6-μm (green) and 10-μm (red) beads spiked in the high-viscosity semen sample during the (**f**) first and (**g**) forth recirculation cycles (scale bar: 200 μm). (**h**) Sperm concentration factor compared to the diluted semen sample prior to the sperm isolation. Comparison of operational performances with DGC; (**i**) recovery of the sperm cell, (**j**) portion of the motile sperm, and (**k**) portion of the dead sperm. The values in graphs of (**h**–**k**) are expressed as the mean ± SD (n = 2–9). WBC, white blood cell; MDDS device, multi-dimensional double spiral device; DGC, density gradient centrifugation.

To increase throughput, we utilized a quad-version device containing four MDDS channels in parallel (Fig. 3a,b). As the sperm cells are collected from the outer wall outlet, the channel configuration of the 3D-printed connector was designed to recirculate and re-process the outer wall output (Fig. 3c and Supplementary Fig. S5). The 3D-printed connector enables direct connection of the MDDS device with syringes (for input and output reservoirs) and check-valves with minimizing undesirable tubing-related dead volumes.

As shown in the Fig. 3d, for the low-viscosity semen sample (with 50 × dilution), 10-μm beads were effectively removed in the first cycle, allowing for recovery of highly purified sperm cells obtained from a total of four cycles of recirculation within 15 min (> 99.95% of 10-μm bead removal and ~ 82% of sperm cell recovery under the optimal flow rate condition, 2.0 × 4 = 8.0 mL/min). On the other hand, in the case of the high-viscosity semen sample (with 50 × dilution), the majority of 10-μm beads (~ 85%) was collected with sperm cells from the outer wall outlet due to the confounding semen secretions in the first separation cycle (Fig. 3e,f). However, the inertial focusing of 10-μm beads returned to the expected behavior as recirculation proceeded due to the removal of the viscous material from the inner wall side outlet in the previous cycles (Fig. 3g). As a result, from the four cycles of recirculation, > 98% of 10-μm beads were removed while keeping sperm cells with a slightly higher recovery rate (~ 87%). Although the removal rate of 10-μm beads is relatively lower for the high-viscosity sample than the low-viscosity sample, the results showed that the recirculation platform could be used as a standardized semen preparation to overcome the variation of fluidic properties caused by the heterogeneity of semen samples.

With each recirculation-separation cycle, sperm cells are recovered into approximately half the input volume, increasing sperm concentration. Considering the variation in the number of sperm cells depending on individual donors, which is in the range of 15–250 × 10^6^ cells/mL for normozoospermic men^43^, we calculated the concentration factor (ratio of the sperm number in the output after separation to the sperm number in the input before separation) instead of the sperm number itself. The results show that sperm cells in the output of ~ 3 mL were approximately tenfold concentrated compared to the input sample of 50 mL within 15 min from 4 recirculation cycles by the recirculation platform having one quad-version MDDS device (1 × MDDS platform) (Fig. 3h). We also tested another recirculation platform having two quad-version MDDS devices (2 × MDDS platform), i.e., eight MDDS devices, where we can achieve higher throughput (faster operation), but the available number of recirculation cycles is reduced from 4 to 3 due to the increased dead volume compared to the 1 × MDDS platform. As a result, due to fewer recirculation cycles, the 2 × MDDS platform brings relatively lower purification and decreased concentrated factor (approximately fivefold), although the operation time was significantly reduced to < 8.5 min, which is much more rapid than the conventional methods (~ 1 h).

The MDDS operation was compared with the DGC method, which is the most standard semen preparation method for ART, in terms of (1) sperm recovery, (2) motility change, and (3) cell viability (Fig. 3i–k). The results from the two MDDS platforms (1 × and 2 × MDDS platforms) were combined into a single plot labeled “MDDS” because there was no significant difference between them in terms of recovery and performance. As shown in Fig. 3i, MDDS shows much higher sperm cell recovery rates overall (approximately 2.7 times higher) than DGC. DGC utilizes sperm density affected by its motility and morphology to isolate just the mature sperm with high motility from a population, whereas MDDS performs sperm isolation in a passive manner by size-based separation utilizing inertial focusing in the device. Therefore, DGC results in low cell number recovery, but the recovered cells are classified as mainly viable (DAPI exclusion) and motile (Fig. 3j,k). In contrast, MDDS provides a high sperm cell recovery with no significant change in overall sperm cell motility and viability compared to the starting sample. The results are in line with the analysis in the previous works that showed no damage induced by the inertial microfluidic separation despite its high flow rate nature^26,27^.

## Discussion and conclusion

The sample-dependent variation in fluidic properties is a major bottleneck for semen preparation by microfluidics. In human sperm purification using inertial microfluidic devices, we can avoid the variation and achieve reliable device performance by using washed seminal cells or washed semen samples after a pre-washing step where the seminal cell pellet was separated from the seminal plasma by centrifugation^17,18,26,27^, but the additional washing step limits the technology’s clinical impact and field deployability. In this work, we developed a recirculating cell sorting platform that can directly process raw semen samples regardless of sample-dependent fluidic conditions only after sample dilution, excluding any additional pre-washing step. Due to the efficient purification and concentration enabled by the recirculation platform, one can still concentrate the sperm in the final purified output (up to tenfold compared with the input sperm concentration). From the recirculation process of the high-viscosity semen sample, we found that initial inertial focusing of 10-μm beads (representing leukocytes) was inhibited as they were collected with sperm cells from the outer wall outlet during the first (1–2) recirculation cycles, but this effect was mitigated as the recirculation process was repeated, resulting in proper separation and removal of 10-μm beads from sperm cells after several cycles. We believe the interference arises from inhomogeneity of the initial semen sample, namely that the viscosity can vary throughout the liquid and even form a loose gel-like matrix. Any gel-like material may be focused to the inner wall side of the second spiral channel in the MDDS device, interfering with the focal position of 10-μm beads. Such an effect is observed to be reduced after a few recirculation cycles, most likely due to homogenization of the sample and removal of the material from the inner wall waste outlet, preventing further interference on 10-μm beads’ focusing to the inner wall side in subsequent passes through the MDDS device. We speculate that seminal and prostate vesicles and their products (fluids, proteins, and complexes), which make up about 95% of semen, are the main components to cause the non-uniform viscosity in the semen and the irregular gel-like matrix in the channels of the MDDS device^44^.

Although the results showed that the recirculating MDDS platform sufficiently removed 10-μm beads regardless of semen’s fluidic conditions, the removal rate was still slightly lower for the high-viscosity samples (> 98%) compared to the low-viscosity samples (> 99.95%). For more consistent outcomes, the operation of the MDDS platform could be further optimized. In the MDDS platform, the final output quality representing recovery, purity, and concentration of sperm cells as well as the operation time is affected by various operational parameters, such as the number of recirculation cycles, initial dilution condition, and the number of MDDS devices used in parallel. An increase of recirculation cycles will offer higher purity and an increase in cell concentration but will lead to lower overall cell recovery while requiring an increased operation time. Higher initial dilutions could mitigate the sample dependency and improve separation performance but require more recirculation cycles with accompanying side effects. As previously discussed in Fig. 3h, the increase in the number of engaged MDDS devices provides higher throughput but increases dead volume and requires a more complex device connection.

We found that sperm cells were not focused sufficiently on the inner-wall side in the first spiral channel of the MDDS device, different from the movement of 6-μm beads in our previous work^32^. The result implies that the effective size of the sperm cells is smaller, due to the alignment of sperm cells along with the direction of the primary flow, their effective size is closer to their head size (~ 5 µm in length, ~ 3 µm in width) rather than their average size (5 μm) which includes their tail length in its calculation^26^. As a result, sperm cells formed a focused band not at the inner wall side but in the middle of the channel after flowing through the first spiral channel. Nevertheless, due to the focusing effect in the first spiral channel, dispersion of particles and cells was significantly reduced, and sperm cells were more efficiently separated from the 10-μm beads (representing leukocytes) with their more focused bands (having a similar location with spheres in the diameter range of 3–6 μm) in the outlet bifurcation region, compared to the single spiral device (Fig. 1c vs. Supplementary Fig. S2b). To achieve better separation performance, the goal of future work is to modify the channel dimensions of the current MDDS device to improve focusing in the first channel.

In addition to high separation efficiency, high throughput is another key advantage of the MDDS separation technology for large sample volume processing, which can solve a critical limitation of other microfluidic sperm separation technologies in terms of their commercialization^9,13,21,22^. Here, we showed that 1 mL of semen could be processed within 8.5 min in the high-throughput fluidic platform, and further throughput increase can be easily achieved by multiplexing or stacking the devices^30,45–48^. The advantage of high throughput could enable its standardized usage in handling semen samples with large variations in their cell number and volume (for normozoospermic men, the sperm count is in the range of 15–250 × 10^6^ cells/mL in 1.5–7.6 mL semen)^43^.

The conventional semen preparation methods such as swim-up and DGC are based on the motility of sperm cells, which significantly limits sperm recovery and avoids recovery of viable but non-motile sperm that are frequently present in testicular sperm extraction^27^. Meanwhile, by using the MDDS separation technology, sperm cells can be collected regardless of their motility and viability due to the nature of its separation principle. We can achieve much higher sperm cell recovery than the conventional centrifugation-based or swim-up methods. The advantage of high sperm cell recovery could lead to a higher success rate of ART, especially IUI, which requires high sperm numbers and concentration^23–25^. In particular, the motility-independent separation capability is beneficial for patients who can produce only immature or low-motility sperm cells and also enables comprehensive analysis of clinical semen samples for diagnostic purposes^17^. In addition, the MDDS separation technology circumvents the need for centrifuging, which is believed to cause damage to the sperm cell. However, we point out that the current MDDS technology offers a relatively lower proportion of motile and viable sperm than conventional methods, limiting its usefulness for certain ART cases where the collection of only “healthy” sperm cells is more desired than the high sperm number. To overcome this limitation, we are planning to advance the MDDS separation device for additional (and optional) selection of healthy sperm by integrating other microfluidic techniques, where motility-based separation can be processed to isolate only healthy and motile sperm in the microfluidic channel, similar to the conventional swim-up method^9,13,21,22,49^.

In summary, the MDDS platform was utilized for sperm sample preparation, with a focus on IUI applications, to replace conventional semen preparation methodologies that have low sperm recovery and operational limitations. The platform showed great separation performance (~ 80% of sperm cell recovery, > 99.95% and > 98% removal of 10-μm beads (a surrogate for leukocytes) from low-viscosity and high-viscosity semen samples, up to tenfold preconcentration of sperm cells compared with the input) and provided critical advantages, including (1) rapid and fully-automated operation (~ 10 min for 50 mL of diluted semen sample) not requiring any pre-washing step, (2) continuous-flow separation (easy output acquisition), (3) minimal cell damage, (4) large sample volume processing (> 1 mL of raw semen), and (5) compact and portable setup. Therefore, we anticipate that the developed platform could be a standardized semen preparation method to achieve pure sperm cells for assisted reproductive technologies (ART) as well as various semen analysis researches.

## Supplementary Information

Supplementary Information.

## Data Availability

The datasets used and/or analysed during the current study available from the corresponding author on reasonable request.
