# Supplementary material for: Multi-dimensional-double-spiral (MDDS) inertial microfluidic platform for sperm isolation directly from the raw semen sample
Source: Sci Rep. 2022 Mar 10;12:4212. doi: 10.1038/s41598-022-08042-1 (PMC8913683; doi:10.1038/s41598-022-08042-1)
Supplement: Supplementary file 1 — Supplementary Information. [file 41598_2022_8042_MOESM1_ESM.docx]

- Supplementary Information -

Multi-dimensional-double-spiral (MDDS) inertial microfluidic platform for sperm isolation directly from the raw semen sample

Hyungkook Jeon,^a^ Claudia Cremers,^d^ Doris Le,^d^ Justin Abell,^d^ and Jongyoon Han^a,b,c,^*

^a^Research Laboratory of Electronics, ^b^Department of Electrical Engineering and Computer Science, and ^c^Department of Biological Engineering, Massachusetts Institute of Technology (MIT), Cambridge, MA 02139, USA.

^d^Ohana Biosciences, 20 Acorn Park Dr., Cambridge, MA 02140, USA.

*Corresponding author: jyhan@mit.edu

Table of Contents

**Fig. S1**. (a) Channel configuration of the five-outlets MDDS device; red-dotted line denotes the observation region. (b) Recoveries from each outlet of 2.1-µm (◼, beige), 3.87-µm (◆, orange), 4.88-µm (⯆, purple), 6-µm (⯅, green), 7.32-µm (◼, red), and 10-µm beads (⚫, blue) at a flow rate of 2 mL/min in the five-outlets MDDS device. (c) Recoveries from each outlet of washed sperm cells (⚫, blue) and PBMCs (◼, red) at a flow rate of 2 mL/min in the five-outlets MDDS device. The recoveries were calculated based on the bead and cell counts measured by flow cytometry. MDDS device, multi-dimensional double spiral device; PBMC, peripheral blood mononuclear cells.

**Fig. S2**. (a) Channel configuration of the single spiral device which has a trapezoidal cross-section with 800 μm in width and 80 and 120 μm in height for the inner wall side and the outer wall side, respectively. (b) Trajectories of sperm cells (green) and 10-μm beads (mimicking leukocytes, red) (scale bar: 200 μm). IW, inner wall; OW, outer wall.

**Fig. S3**. Visualization of input and output samples by CASA (computer-assisted sperm analysis), which shows the separation of 10-μm beads and sperm cells. IW, inner wall; OW, outer wall.

**Fig. S4**. An example of forward and side scatter plots in flow cytometry, depicting the sperm cells (red) and debris (blue).

**Fig. S5**. (a) Photos of the 1×MDDS platform and its experimental setup. (b) Photos of the 2×MDDS platform and its experimental setup. MDDS device, multi-dimensional double spiral device.


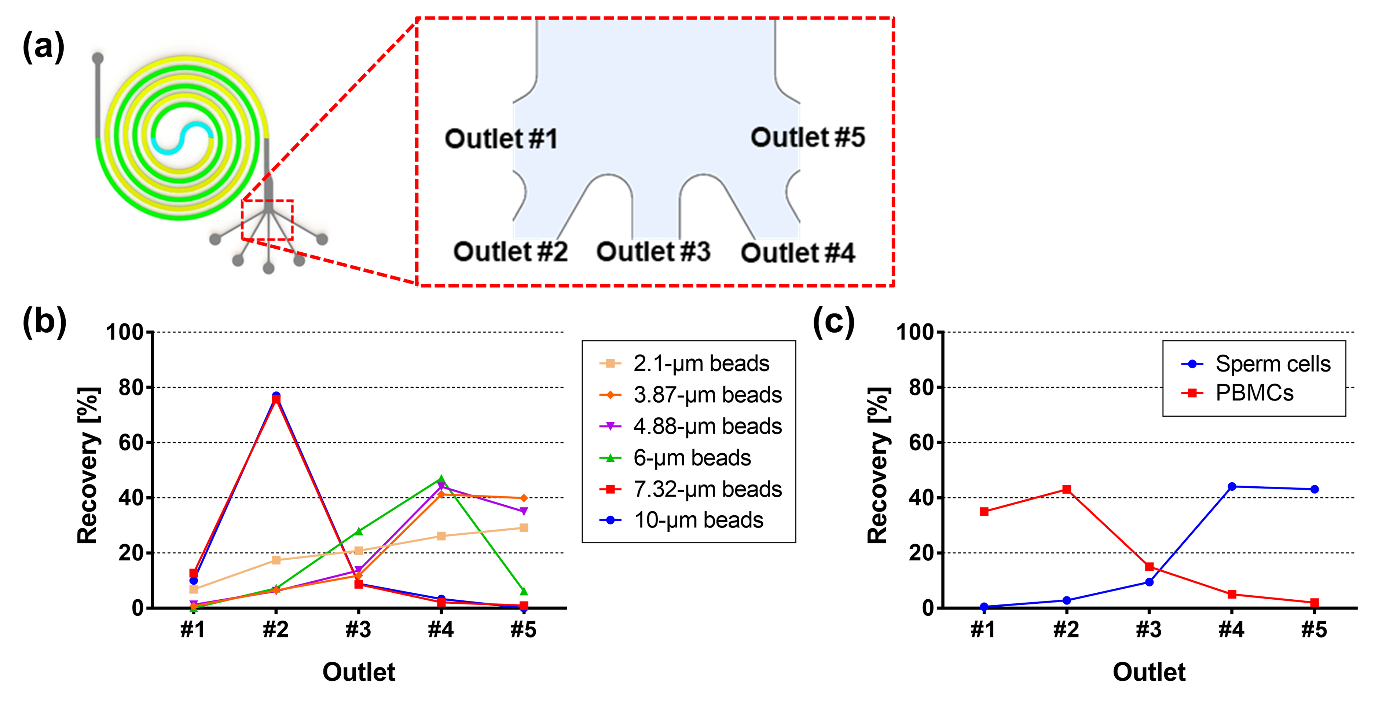


**Fig. S1**. (a) Channel configuration of the five-outlets MDDS device; red-dotted line denotes the observation region. (b) Recoveries from each outlet of 2.1-µm (◼, beige), 3.87-µm (◆, orange), 4.88-µm (⯆, purple), 6-µm (⯅, green), 7.32-µm (◼, red), and 10-µm beads (⚫, blue) at a flow rate of 2 mL/min in the five-outlets MDDS device. (c) Recoveries from each outlet of washed sperm cells (⚫, blue) and PBMCs (◼, red) at a flow rate of 2 mL/min in the five-outlets MDDS device. The recoveries were calculated based on the bead and cell counts measured by flow cytometry. MDDS device, multi-dimensional double spiral device; PBMC, peripheral blood mononuclear cells.


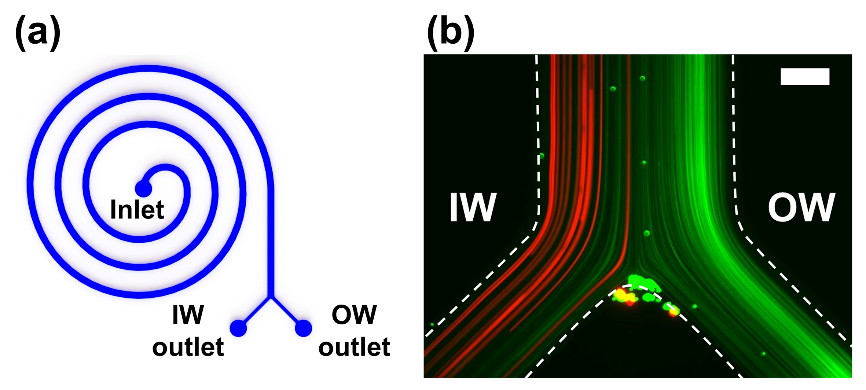


**Fig. S2**. (a) Channel configuration of the single spiral device which has a trapezoidal cross-section with 800 μm in width and 80 and 120 μm in height for the inner wall side and the outer wall side, respectively. (b) Trajectories of sperm cells (green) and 10-μm beads (mimicking leukocytes, red) (scale bar: 200 μm). IW, inner wall; OW, outer wall.


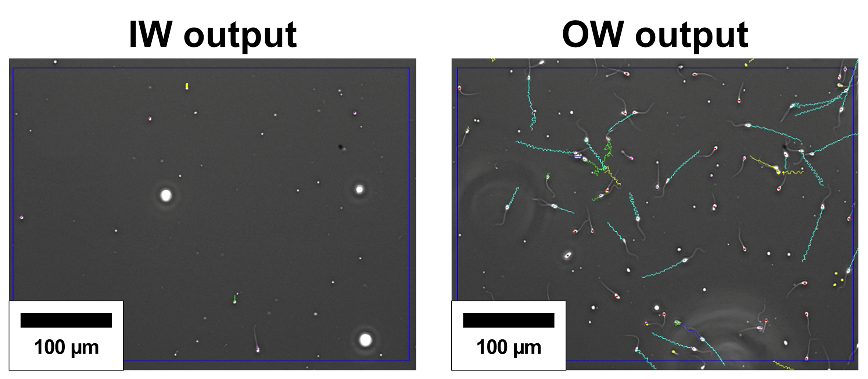


**Fig. S3**. Visualization of input and output samples by CASA (computer-assisted sperm analysis), which shows the separation of 10-μm beads and sperm cells. IW, inner wall; OW, outer wall.


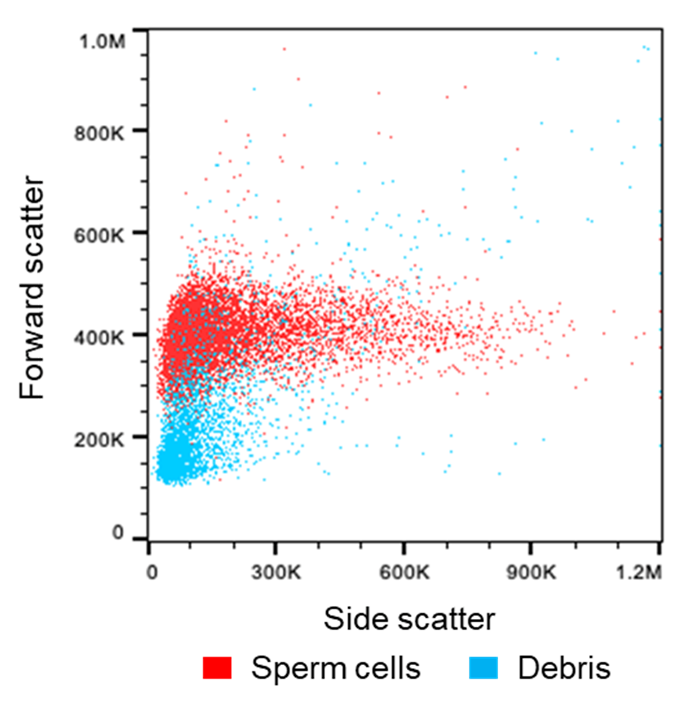


**Fig. S4**. An example of forward and side scatter plots in flow cytometry, depicting the sperm cells (red) and debris (blue).


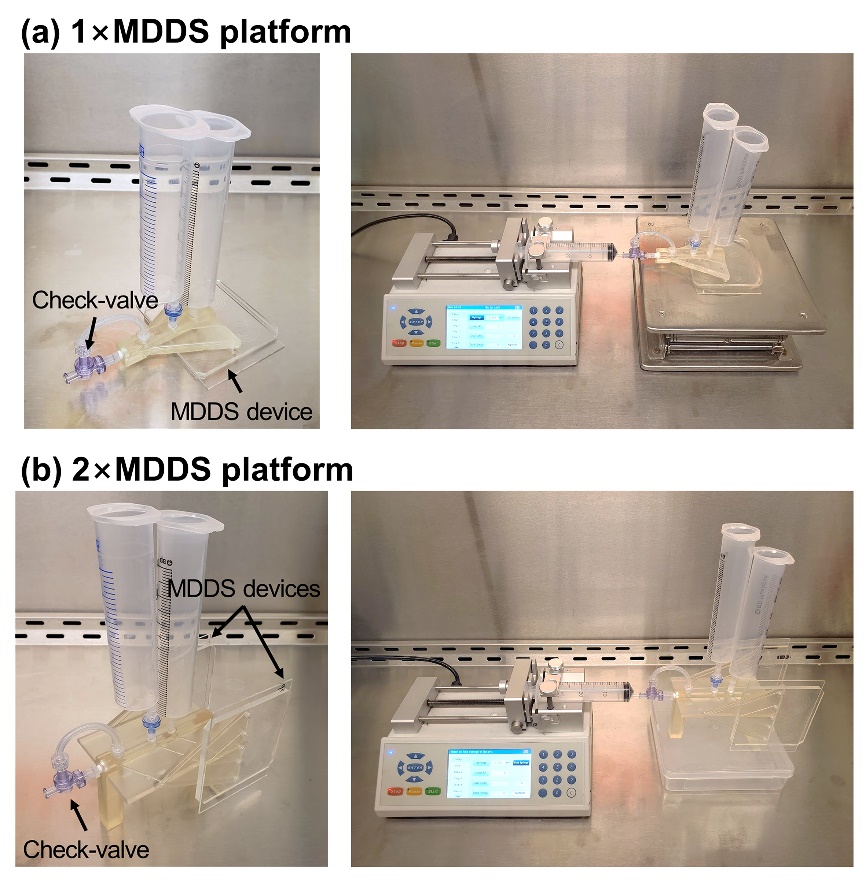


**Fig. S5**. (a) Photos of the 1×MDDS platform and its experimental setup. (b) Photos of the 2×MDDS platform and its experimental setup. MDDS device, multi-dimensional double spiral device.
